# Supplementary material for: The causal role of circulating inflammatory markers in osteoporosis: a bidirectional Mendelian randomized study
Source: Front Immunol. 2024 Jul 18;15:1412298. doi: 10.3389/fimmu.2024.1412298 (PMC11291241; doi:10.3389/fimmu.2024.1412298)
Supplement: Supplementary file 1 [file DataSheet_1.zip › supplementary material/OSTEOPOROSIS-CIM/GCST90274826.tsv.gz_leave_one_out.pdf]

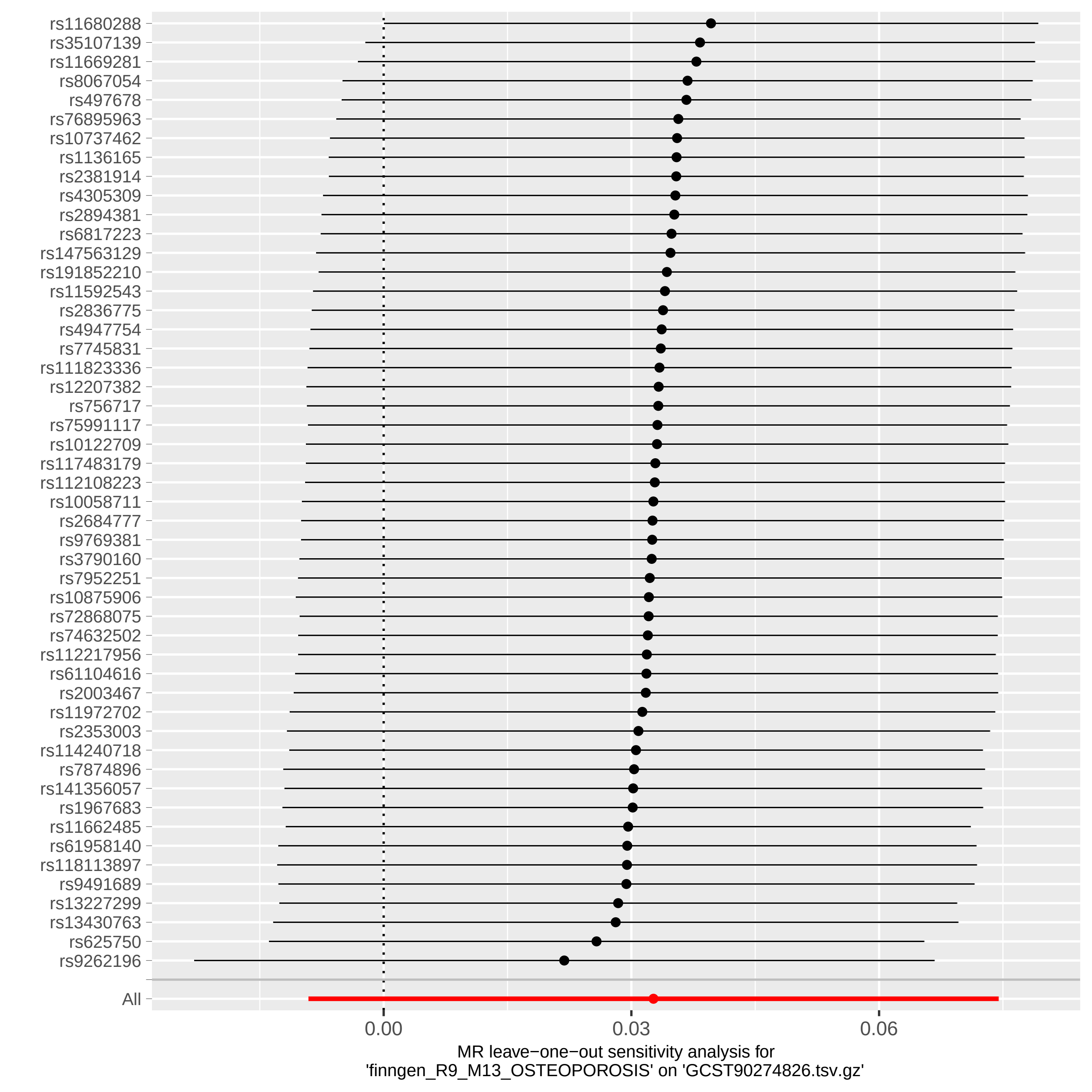
rs11680288
rs35107139
rs11669281
rs8067054
rs497678
rs76895963
rs10737462
rs1136165
rs2381914
rs4305309
rs2894381
rs6817223
rs147563129
rs191852210
rs11592543
rs2836775
rs4947754
rs7745831
rs111823336
rs12207382
rs756717
rs75991117
rs10122709
rs117483179
rs112108223
rs10058711
rs2684777
rs9769381
rs3790160
rs7952251
rs10875906
rs72868075
rs74632502
rs112217956
rs61104616
rs2003467
rs11972702
rs2353003
rs114240718
rs7874896
rs141356057
rs1967683
rs11662485
rs61958140
rs118113897
rs9491689
rs13227299
rs13430763
rs625750
rs9262196
All
0.00
0.03
0.06
MR leave−one−out sensitivity analysis for
'finngen_R9_M13_OSTEOPOROSIS' on 'GCST90274826.tsv.gz'
